# A total laparoscopic treatment strategy for Amyand’s hernia complicated with appendicitis: A case report

**DOI:** 10.1016/j.ijscr.2019.04.049

**Published:** 2019-05-07

**Authors:** Shen-Hung Han, Ming-Yi Li, Hung-Fei Lai

**Affiliations:** Division of General Surgery, Department of Surgery, Mackay Memorial Hospital, #92, Section 2, Chung-San North Road, Taipei, Taiwan

**Keywords:** TEP, total extraperitoneal approach, TAPP, transabdominal preperitoneal approach, SSI, surgical site infection, Amyand’s hernia, Acute appendicitis, Laparoscopic appendectomy, Laparoscopic hernioplasty, Case report

## Abstract

**Introduction:**

The presence of a vermiform appendix in an inguinal hernia sac is known as Amyand’s hernia. It is even rarer with complicated appendicitis. Formerly it was treated via an open groin approach; recently there are new trends involving the use of laparoscopy for reduction and management of appendix. Conversely the role of laparoscopy in the following hernia repair was less discussed, with no standard care. We reported a case of Amyand’s hernia complicated with appendix which was managed via a total laparoscopic strategy.

**Presentation of case:**

A 49-year-old male presented with right groin mass with progressive pain for 3 days. Physical exams revealed incarcerated right inguinal hernia. Amyand's hernia with acute appendicitis was diagnosed preoperatively via computed tomography (CT). Emergent diagnostic laparoscopy was performed. Appendix was reduced, with appendectomy justified for signs of appendicitis. An interval total extraperitoneal (TEP) hernioplasty was performed 3 month later, with no adverse events postoperatively.

**Discussion:**

In virtue of previous literature, we proposed a total laparoscopic strategy for Amyand’s hernia which consists of transabdominal diagnostic laparoscopy, management of appendix, and either immediate or elective laparoscopic hernioplasty, based on status of appendix.

**Conclusion:**

This laparoscopic treatment strategy is feasible for Amyand’s hernia, with minimized risk of surgical site infection (SSI), fair recovery and cosmesis.

## Highlights

• An appendix incarcerated in the inguinal hernia is defined as Amyand’s hernia.
• Preoperative diagnosis of Amyand’s hernia is feasible with ultrasound and CT.
• Laparoscopy for diagnostic and therapeutic purposes has been on an upward trajectory.
• A potential total laparoscopy treatment strategy for Amyand’s hernia was proposed, with fair outcomes.

## 1 Introduction

The presence of the vermiform appendix incarcerated in the inguinal hernia sac was first described by Claudius Amyand in 1735. Incidence of Amyand’s hernia is approximately 1% of inguinal hernias, and less than 0.1% of these cases are complicated with acute appendicitis [[1], [2], [3], [4], [5], [6], [7]]. The clinical presentation of Amyand’s hernia is indistinguishable from an incarcerated hernia. Thus in previous reports diagnosis was usually made intraoperatively [1,2]. However, the widespread use of image tools such as ultrasound and computed tomography (CT) changes the circumstances and aids in preoperative diagnosis [3,4,6]. The precise preoperative diagnosis facilitates a shift in surgery, from an open groin incision to laparoscopic approach. Most recent literature involve the use of laparoscopy for diagnostic and therapeutic purposes, including laparoscopic reduction of appendix and appendectomy if signs of inflammation or perforation [[3], [4], [5], [6]]. In contrast, the role of laparoscopy in the ensuing hernia repair is less discussed except few reports, and there is no standard surgical care [5,[2], [3], [4], [5], [6], [7]]. Therefore, we report a case of Amyand’s hernia complicated with acute appendicitis which was treated by laparoscopic appendectomy followed by elective laparoscopic hernioplasty. Besides, we try to elucidate a total laparoscopic management strategy, thanks to the existing literature. This work has been reported in line with the SCARE criteria [8].

## 2 Case presentation

**Fig. 1:**
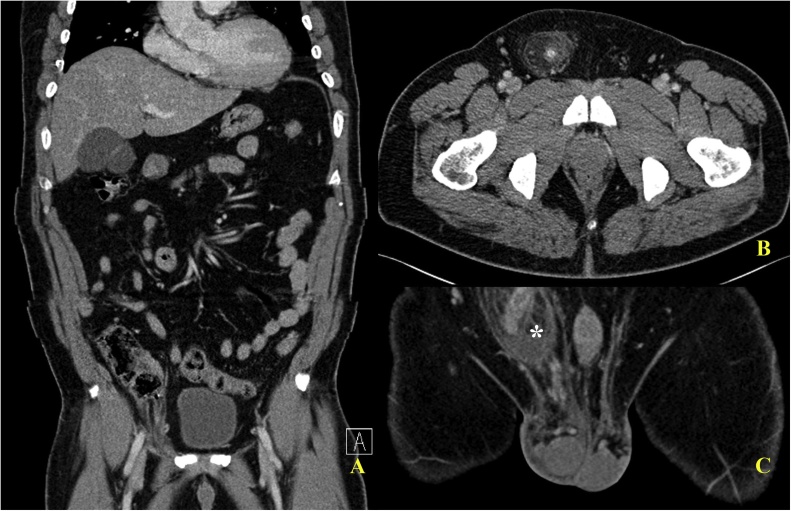
Contrast-enhanced CT images. (A) A blind-ended tubular structure incarcerated into the right inguinal canal (circle). (B) The incarcerated appendix contained an appendocolith. (arrow) (C) Inflamed appendix with perifocal stranding (asterisk).

**Fig. 2:**
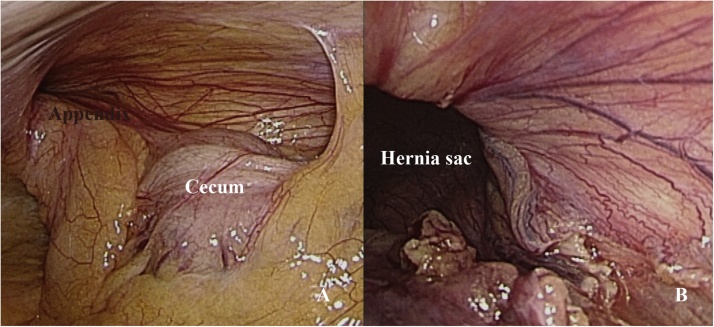
Laparoscopic findings (A)An appendix incarcerated within the right inguinal canal; (B) After laparoscopic lysis of adhesions, the appendix was reduced, with no residual component left in the sac.

A 49-year-old male patient arrived at our emergency department with a painful right inguinal mass. The patient has unremarkable past medical and surgical history. He was afebrile, with vital signs within normal limits. The patient presented with a reducible right inguinal mass in the preceding 6 months, which became irreducible 3 days ago, with associated localized pain. The nonreducible right groin mass persisted, and the pain progressed. Manual reduction failed after moderate sedation. Laboratory tests revealed leukocytosis (12,800 white blood cells/μL). Contrast-enhanced CT of the abdomen and pelvis was performed. The images demonstrated that a dilated appendix with appendicolith and perifocal fat stranding in the right inguinal canal (Fig. 1A–C). A preoperative diagnosis of Amyand’s hernia was highly suspected. After obtaining informed consent, diagnostic transabdominal laparoscopy was performed which disclosing an engorged appendix incarcerated in the right inguinal internal ring (Fig. 2A). After laparoscopic lysis of the adhesions, the appendix was reduced. The hernia sac was checked with no residual component (Fig. 2B). Because acute appendicitis seemed to be a straightforward diagnosis grossly without suspicion, laparoscopic appendectomy was performed. Pathological examination confirmed acute appendicitis, with fibrinopurulent exudate and a 0.3 cm fecalith impaction. Concerning about the infectious complications of immediate mesh repair, we decided to perform hernia repair in an elective setting, that is, after resolution of localized infection and inflammation. Postoperative course was smooth, and the patient was discharged 2 days later. Elective laparoscopic total extraperitoneal (TEP) hernioplasty with 10 × 15 cm polypropylene mesh was performed 3 months later. No adverse events occurred postoperatively.

## 3 Discussion

Amyand’s hernia is an eponymous and rare condition. It combines two common situations of general surgery. Both situations merit consideration and being treated as a whole [1]. The inflammatory status of the appendix in the hernia sac dictates the surgical options [2]. Losanoff and Basson proposed a classification system which divided Amyand’s hernia into four subtypes. Each subtype is treated differently, mainly by open surgery [1].

The operative approach was known historically as via a groin incision or laparotomy; recently there are increasing reports emphasizing the use of laparoscopy for both diagnostic and therapeutic purposes [[3], [4], [5], [6], [7]]. The change in surgical approach is owing to the widespread use of ultrasound and CT which makes preoperative diagnosis feasible. Vermillion et al. reported the first laparoscopic reduction of Amyand’s hernia in 1999. The acute inflamed appendix was then removed laparoscopically, followed by an elective Lichtenstein repair [3]. The role of laparoscopy in diagnosis, reduction, and managing appendix stump was verified by subsequent reports [4,6]. The laparoscopic transabdominal approach provides the advantage of better visualization and management of the whole appendix and its base. Besides, sac contents can be reduced safely under direct visualization [4].

On the other hand, few reports discussed about the role of laparoscopy for the following hernia repair. The surgical approach varied among reports, and there remained no standard surgical care. It is well recognized that if the appendix is normal looking or untraumatized, appendectomy is not recommended [1,2,7]. Therefore concurrent mesh hernioplasty after diagnostic laparoscopy is an appropriate option. Sahu et al. described three cases of laparoscopic transabdominal preperitoneal hernioplasty (TAPP) without appendectomy, given that appendix found to be grossly normal [7].

When encountering an inflamed or perforated appendix in Amyand’s hernia necessitating appendectomy, controversy exists regarding the method of hernia repair especially the use of mesh. Saggar et al. described endoscopic total extraperitoneal approach, with appendectomy and mesh hernioplasty in preperitoneal space [5]. Mullinax et al. reported a case with simultaneous laparoscopic appendectomy and inguinal hernia repair [6]. We assumed it is technically more difficult to manage appendix base in extraperitoneal approach than transabdominal approach; while the latter did not mention the manner of laparoscopic hernia repair in context. Both pose the concern of SSI when performing immediate mesh repair in a contaminated wound. However, the mesh repair technique is a good option for the treatment of strangulated inguinal hernias in adults, giving an acceptable wound infection rate and fewer recurrences than non-mesh repair [9]. In this situation, we advocated an elective laparoscopic mesh hernioplasty, namely interval or two-stage approach, after resolution of localized inflammation and infection. The role of two-stage surgery in minimizing postoperative SSI is highlighted by Akaishi et al., yet open inguinal approach was used [4]. In our case, appendectomy is indicated for gross and radiological evidence of appendicitis. Thus, we performed two-stage surgical approach including laparoscopic appendectomy and elective laparoscopic TEP repair. TAPP hernioplasty might be used equivalently on surgeon preference.

**Fig. 3:**
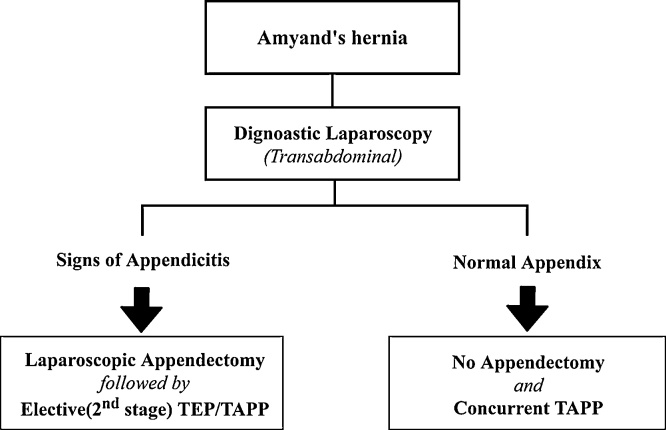
A total laparoscopic treatment strategy for Amyand’s hernia.

Incorporating the aforementioned concepts, we proposed a total laparoscopic treatment strategy for Amyand’s hernia (Fig. 3). Transabdominal diagnostic laparoscopy serves as the first step, with lysis of adhesion and reduction of appendix carefully. The decision to perform appendectomy or not should be made in light of laparoscopic finding. Then immediate or interval mesh hernioplasty might be determined, based on whether appendectomy or not. The prerequisite of this laparoscopic strategy is that preoperative diagnosis must be made precisely by ultrasound or CT, as previous reports [3,4,6]. Certainly the alternative treatment is elective Lichtenstein repair after laparoscopic appendectomy, as the initial report of Vermillion et al. [3]. Nevertheless, it is justified to address this entity with a total laparoscopic approach in an era of minimal invasive surgery. The transabdominal laparoscopic approach has the benefit of easier reduction of appendix and less degree of anatomical structure weakening by not enlarging the hernia defect and neck of sac [4]. The use of laparoscopy also reduces the chance of adhesion which renders the second stage laparoscopic hernioplasty a feasible and rational option. Besides, faster recovery and better cosmesis are anticipated.

In conclusion, a total laparoscopic strategy consisting of diagnostic laparoscopy, management of appendix, and mesh hernioplasty is feasible for patients with Amyand’s hernia. The outcome, morbidity, and cosmetic results are satisfactory.

## Conflict of interest

All authors have no conflicts of interest to declare.

## Funding

The authors declare no funding or sponsorship.

## Ethical approval

Ethical approval has been obtained from the institutional review board in MacKay Memorial Hospital.

## Consent

The authors have obtained written informed consent from the patient for publication of this case report and accompanying images.

## Author’s contribution

Shen-Hung Han wrote the original draft and edited the manuscript. Ming-Yi Li performed the surgery and conceptualized the strategy. Hung-Fei Lai supervised the draft and revised the manuscript.

## Registration of research studies

Not applicable.

## Guarantor

Dr. Hung-Fei Lai.

## Provenance and peer review

Not commissioned, externally peer-reviewed.

## References

[1] Michalinos A., Moris D., Vernadakis S. (2014). Amyand’s hernia: a review. Am. J. Surg..

[2] Sharma H. (2007). Amyand’s hernia: a report of 18 consecutive patients over a 15-year period. Hernia.

[3] Vermillion J.M., Abernathy S.W., Snyder S.K. (1999). Laparoscopic reduction of Amyand’s hernia. Hernia.

[4] Akaishi R. (2018). Amyand’s hernia complicated with appendix perforation treated by two-stage surgery consisting of laparoscopic appendectomy followed by elective inguinal hernioplasty: a case report. Int. J. Surg. Case Rep..

[5] Saggar V.R., Singh K., Sarangi R. (2004). Endoscopic total extraperitoneal management of Amyand’s hernia. Hernia.

[6] Mullinax J.E., Allins A., Avital I. (2011). Laparoscopic appendectomy for Amyand’s hernia: a modern approach to a historic diagnosis. J. Gastrointest. Surg..

[7] Sahu D. (2015). Amyand’s hernia: our experience in the laparoscopic era. J Minimal Access Surg..

[8] Agha R.A., Borrelli M.R., Farwana R., Koshy K., Fowler A., Orgill D.P., For the SCARE Group (2018). The SCARE 2018 statement: updating consensus Surgical CAse REport (SCARE) guidelines. Int. J. Surg..

[9] Hentati H.1, Dougaz W., Dziri C. (2014). Mesh repair versus non-mesh repair for strangulated inguinal hernia: systematic review with meta-analysis. World J. Surg..

